# Correction to “Inhibition of the Numb/Notch Signaling Pathway Increases Radiation Sensitivity in Human Nasopharyngeal Carcinoma Cells”

**DOI:** 10.1002/kjm2.70153

**Published:** 2025-12-26

E.‐D. Shen and Q. Zeng, “Inhibition of the Numb/Notch Signaling Pathway Increases Radiation Sensitivity in Human Nasopharyngeal Carcinoma Cells,” *Kaohsiung Journal of Medical Sciences* 35 (2019): 474–485, https://doi.org/10.1002/kjm2.12087.

In our published article “Inhibition of the Numb/Notch signaling pathway increases radiation sensitivity in human nasopharyngeal carcinoma cells”, we identified an inadvertent image duplication in Figure 5A due to a typesetting or figure assembly error. To ensure the accuracy and integrity of the scientific record, we have replaced Figure 5A with the correct image. This correction does not affect any of the study's conclusions or data interpretation, as the revised image accurately represents the original experimental results.

Correct image


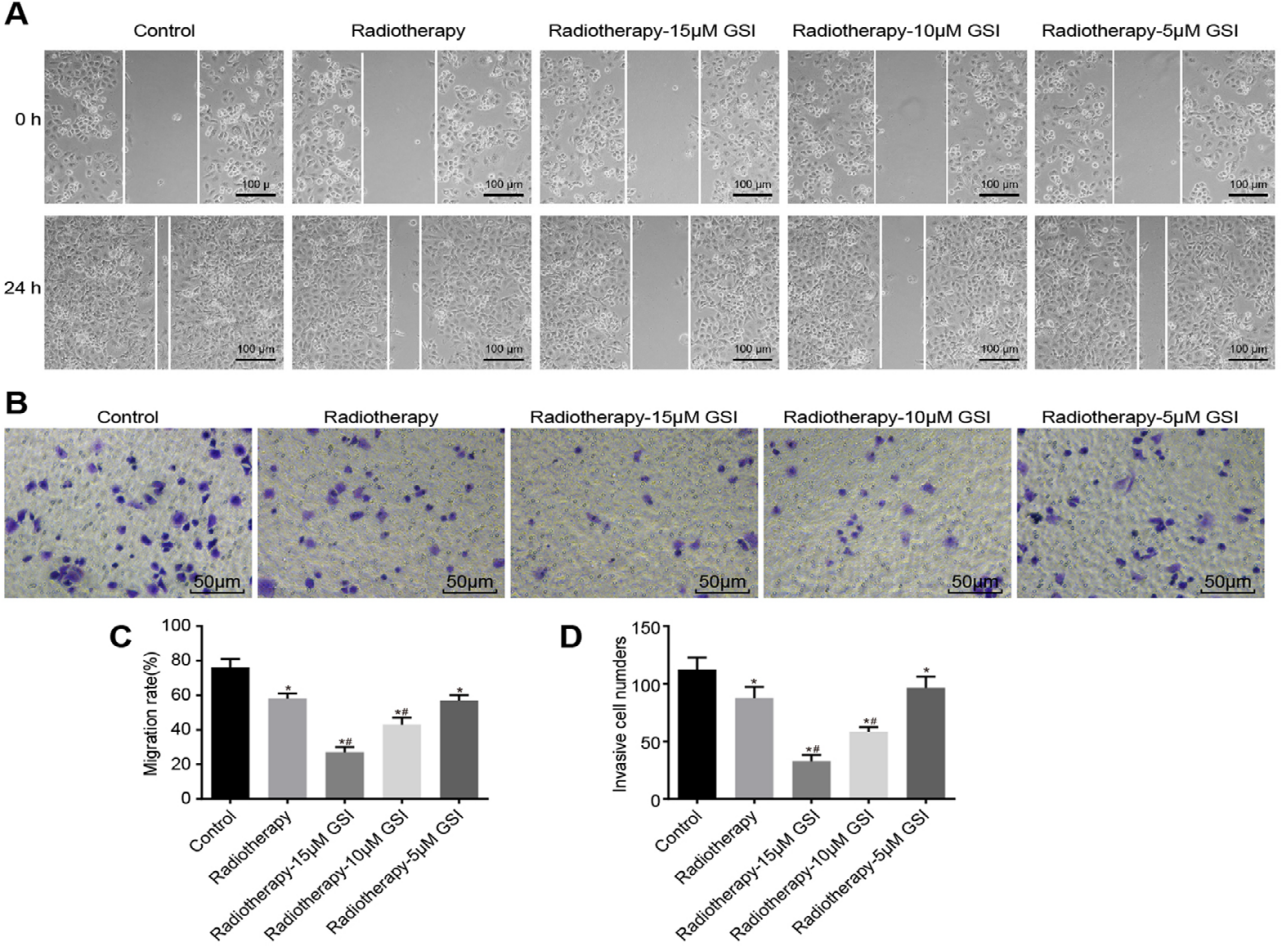


The authors confirmed that all results and conclusions of this article remain unchanged.

We apologize for this error.

